# What the public wants to know about the recycling of contaminated soil

**DOI:** 10.1371/journal.pone.0331478

**Published:** 2025-09-04

**Authors:** Stephen Takeshi Terada, Hitomi Matsunaga, Aizhan Zabirowa, Tomoko Watanabe, Yuya Kashiwazaki, Makiko Orita, Thierry Schneider, Noboru Takamura

**Affiliations:** 1 Department of Global Health, Medicine and Welfare, Atomic Bomb Disease Institute, Nagasaki University, Sakamoto, Nagasaki, Japan; 2 Nuclear Protection Evaluation Centre (CEPN), Fontenay-aux-Roses, France; University of Florida, UNITED STATES OF AMERICA

## Abstract

After the Fukushima Daiichi Nuclear Power Plant accident of March 11, 2011, the Japanese government implemented a soil decontamination program as part of disaster area recovery. This resulted in approximately 14 million cubic meters of contaminated soil being stored in an interim facility in Fukushima. Management of the soil has included radioactivity measurement, separation and screening. When recycled the soil may be used in public works projects, but the public objected to soil recycling demonstration projects planned for the Tokyo metropolitan area, and the use of recycled soil in their communities. This study seeks to understand key factors associated with the public’s desire for more information about soil recycling. We conducted a nationwide survey and received 5257 responses from people between the ages of 18 and 89 living in the country’s 47 prefectures and eight geographic regions. The results showed that approximately 60% of residents did not want recycled soil to be relocated near where they live. A large number (75.9%, n = 3991) of respondents had never received information on radiation and its health effects, while 66.1% (3473) of respondents wanted more information about recycled soil. Those who wanted more information about soil recycling prioritized learning of its health effects, effects on food and water, environmental effects, radioactivity levels and monitoring methods, and intergenerational health effects. A binary logistic regression analysis clarified several independent factors related to wanting more information, including interest in the recovery of the area surrounding the FDNPP, receipt of information on radiation and its health effects, and the belief that the FDNPP accident will result in intergenerational health effects. The study results provide a greater understanding of the public’s information needs and can help to improve communication and understanding related to this controversial topic.

## Introduction

The March 2011 nuclear accident at the TEPCO Fukushima Daiichi Nuclear Power Plant (FDNPP) caused widespread radiation contamination in Japan’s Fukushima Prefecture. Between 2014 and 2018, the government conducted primary area decontamination efforts in inhabited areas to remove radioactive materials, which included removing the top layer of contaminated soil from large areas throughout the prefecture. The designated difficult-to-return zone which describes the restricted areas in the Hamadori or coastal region where the FDNPP is located and where residents are not allowed to return because of the high radiation levels from the nuclear accident, were not included in the original decontamination program. However, since 2018 that zone has been undergoing decontamination in certain places to support the return of residents. Decontamination was effective, reducing radiation air dose rates by 60% in residential areas [1]. The removed soil was temporarily stored in local neighborhoods and then transported via truck to a 1,600-hectare interim storage facility (ISF) that straddles Okuma and Futaba towns that are also the municipalities where the FDNPP is situated. Construction of the ISF began in November 2016, soil separation activities began the summer of 2017, and after the completion of soil storage facilities the storage of the soil commenced in October 2017 in Okuma and December 2017 in Futaba. According to the Ministry of Environment (MOE), almost all of the removed soil, except for the restricted areas in Hamadori, was moved to the ISF by March 2022 [2]. A very large amount of contaminated soil – approximately 14 million cubic meters that was previously measured for radioactivity, separated and screened [3] – is now being managed and stored at the ISF. According to national law, the stored soil must be relocated from the ISF to a permanent site outside of Fukushima Prefecture by 2045 [2]. For perspective, no other radiation accidents have required the collection, movement, storage, and final disposal of this amount of contaminated soil.

National legislation enacted between 2011 and 2019 progressively defined the Japanese government’s plan for the soil’s disposal. The laws stipulate that the soil will be managed and its volume reduced; the national government would be the responsible body; soil recycling will be promoted, with disposal outside of the Fukushima Prefecture; and, finally, that the government would reduce the amount of soil to be recycled and secure the understanding of local communities [1]. A report issued in September 2024 by the International Atomic Energy Agency (IAEA) stated that its 16-month-long review of Japan’s approach to Fukushima soil management and recycling met IAEA safety standards [3]. The IAEA report noted that the soil management program followed Japanese law that permits soil repurposing in Japan, and that the government’s intent was to recycle about 75% of the stored soil that had low radioactivity levels and when proven to be safe, into projects such as seawalls, agricultural land, road embankments, waste treatment sites, railways, and coastal protection. Soil that could not be recycled would be permanently disposed of, and that the government intended to identify both site selection and disposal processes in 2025 [3].

The recycling of soil to be used in public works projects follows the MOE’s June 2016 Basic Concept that sets out the proper use of removed soil after the soil volume has been reduced as a basic requirement for radiation safety [2]. Under the Basic Concept, MOE carries out demonstration projects while confirming radiation safety, evaluates management systems, promotes public understanding of soil recycling, and moves toward comprehensive soil recycling and the improvement of the environment. The use of contaminated soil would be limited to public projects where management responsibilities and physical properties were clearly established, such as for an embankment whose structure and shape would not change for a long time [2]. Proper management under the Basic Concept limits the recycle projects’ additional radiation exposure doses for construction workers to below 1 millisievert per year (mSv/year) during construction and repair, and below 0.01 mSv/year of additional radiation exposure to members of the public while in use [4,5]. The maximum soil recycling radioactivity level is below 8,000 becquerels per kilogram (Bq/kg) and is determined in accordance with its use [2]. Shielding will be used to cover the recycled soil and to prevent scattering and leakage, and the project data is to be recorded. Implementation of the soil recycling policy began with the MOE’s environmental regeneration demonstration project at Iitate Village, in the Fukushima Prefecture, in 2017, where contaminated soil was buried under agricultural fields to confirm safety and soil productivity, and to test cultivation of crops. The MOE has reported that radiation monitoring indicated no safety concerns for the public [2] due to the harvested crops at Nagadoro District in 2020 and 2021 showing radioactive cesium levels of 0.1–2.5 Bq/kg, far below the Japan standard limit of 100 Bq/kg for general foods [6].

The MOE planned another demonstration project underneath pathways adjacent to rice fields in Nihonmatsu City, Fukushima, but residents there objected [7] and the project was cancelled in 2018. The residents of Nihonmatsu voiced multiple concerns [7], such as “Our biggest concern was there’s no guarantee the road won’t crumble during heavy rain, or in another earthquake…rain will soak the ground and besides this road, there’s a river that carries water for farming – for the rice fields, a town, and a school. We’re worried the contaminated water would leak.” Also comments such as “Who decided to hold the tests in Nihonmatsu?” and “If the soil is safe, why not use it for public works construction projects for the Tokyo Olympics?”

Strong public objections to soil recycling demonstration projects spotlight the well-known concerns of people who are confronted with non-radioactive hazardous waste storage near the areas where they live [8]; in this instance, residents have the added burden of assuming the risk of having radiation-contaminated waste stored in the form of recycled soil where they live [9]. Several studies completed in 2022 examined the public’s acceptance of the disposal of contaminated soil outside of the Fukushima Prefecture from different perspectives [10]. Takada et al. found significant public support for soil disposal outside of Fukushima; however, the public was not well-informed about the government’s disposal policy and disagreed with policy implementation that was viewed as top-down and demonstrating insufficient information disclosure and clarity – both of which are key elements of procedural fairness. Those who did not accept soil disposal outside of Fukushima did not agree that such disposal was necessary, and they were unhappy with the perceived lack of transparency and disclosure of information. Shirai et al. [11] found that trust in the MOE and local municipalities had a significant effect on the acceptance or rejection of soil disposal in residential neighborhoods, and that it had a significant effect on all the factors in their study resulting from what they termed “indirect effects.” Yokoyama et al. [12] noted that, when nuclear waste candidate sites were decided in advance without citizens’ input, the public was mistrustful of the process.

Previous studies have examined the importance of trust, transparency, and information disclosure but have not clearly indicated what information is required. The purpose of this study was to clarify the information that is useful for understanding soil recycling based on residents’ perceptions of radiation and their concerns about the Fukushima Daiichi nuclear accident. The results of this study provide insights into the information that the general public is looking for regarding soil recycling.

## Materials and methods

### Participants

The survey was conducted from May 27–29, 2024 among participants registered with the online survey company Cross Marketing, Inc., which was contracted for this study, and included only those registrants who had signed a confidentiality agreement. Survey responses were collected through a web survey and adjusted by gender and age group according to the respective population demographics of the region surveyed. This study is based on national survey responses received from residents in the Fukushima Prefecture and Japan’s eight regional areas – Hokkaido, Tohoku, Kanto, Chubu, Kinki, Chugoku, Shikoku, and Kyushu/Okinawa. The regional areas cover all of Japan, and the data were provided for regional characteristics. All survey respondents were aged 18 or older. A final sample of 5257 survey responses was received for this study.

### Questionnaire

The questionnaire used in this study was developed based on a survey in previous studies conducted in the Fukushima Prefecture regarding the information [13,14]. The questionnaire for this study consisted of information transmission and its desires. The survey items included demographic information such as current residential area, sex, and age. Answers to two questions related to gathering information and attending lectures about radiation and its health effects were answered with either “yes” or “no.” For those who responded “yes” about gathering information, there were nine allowed responses, with multiple choices permitted: internet search engines; TV programs/newspapers; news sites; books and magazines; parents/friends/teachers, and acquaintances; social networking sites; video streaming services; computer applications; and other. We also asked whether respondents were interested in topics related to the disaster and the environment. The survey inquired about the risk perception of intergenerational radiation health effects stemming from the nuclear accident, whether respondents would like to visit the area around the FDNPP and also gauged their interest in the disaster recovery efforts. Two main questions addressed the recycling of soil generated from the decontamination works: whether respondents would accept the reuse of the soil in their current residential area, and whether they would like more information on it. In these questions asked risk perception or recognition, four responses were possible: yes, probably yes, probably no, and no. Moreover, respondents were asked what they wanted more information about, regarding the recycling of removed soil in their current residential area. For this question, they could select multiple responses, which included ones concerning health effects, effects on food and water, environmental effects, radioactivity concentration, monitoring methods, intergenerational health effects, information disclosure methods, risks during disasters, recycling methods, and other information. (S1 Appendix).

### Ethical considerations

The study was approved by the ethics committee of the Nagasaki University Graduate School of Biomedical Sciences (No. 24042603). The study was also conducted in accordance with the *Ethical Guidelines for Life Science and Medical Research Involving Human Subjects* set forth by Japan’s Ministry of Education, Culture, Sports, Science and Technology and the Japanese Ministry of Health, Labor, and Welfare. The purpose, methods, and ethical considerations of the study were explained on the website’s opening page, and responses to the questionnaire provided informed consent.

### Statistical analysis

To compare related factors for wanting more information about the recycling of soil generated from the FDNPP, the answers were divided into two categories: “yes” and “probably yes,” as “YES,” and “no” or “probably no,” as “NO.” Respondents’ ages were combined and analyzed by decade, apart from “teens and 20s” and “80s and 90s” as combined cohorts, based on the research cohort database. The study used chi-square tests to analyze the sources of information participants had received on radiation and its health effects, the information they required for an informed decision on the reuse of recycled soil in the areas where they live, and the relationship between these factors and the desire for information about soil recycling. Then, the independently associated factors for wanting more information about soil recycling were evaluated using binary logistic regression analysis. After excluding statistically confounding factors, the model was selected and calculated from the significant factors revealed in the chi-square test. The regression model of wanting more information about soil recycling had yes as a “reference” and calculated odds ratios (OR), 95% confidence intervals (95% CI), and *p*-values. The analysis was performed using IBM SPSS Statistics Version 29.0. software (SPSS Japan, Tokyo, Japan), and statistical significance was set at *p* < 0.05.

## Results

### Respondents’ characteristics

Out of a total 5257 respondents, 1131 responded “yes” (21.5%), 2342 said “probably yes” (44.6%), 1034 answered “probably no” (19.7%), and 750 replied “no” (14.2%) to the question about wanting more information about soil recycling resulting from the FDNPP accident. Hence, those defined as YES for wanting more information accounted for 3473 (66.1%) participants, while NO accounted for 1784 (33.9%) of them. Almost two-thirds of survey respondents wanted more information about soil recycling.

Table 1 shows a comparison of each factor related to wanting more information about recycled soil generated from the FDNPP accident. There were 4523 (86%) survey respondents from the eight regional areas of Japan and an additional 734 (14%) from the Fukushima Prefecture. The eight regions each accounted for 10–12% (n = 523–633) of the survey responses. The survey’s respondents were roughly evenly split by gender, with 2745 males (52.2%) and 2512 females (47.8%). In none of the eight geographic regions did current residential area or sex show a significant difference in participants’ desire for more information on recycled soil, though respondents in the Fukushima Prefecture were an exception, as these rates were statistically significant there (p = 0.002).

**Table 1:** Comparison of each factor related to a desire for more information about recycled soil generated from the FDNPP accident.

	Response	Overall N = 5257	Yes	No	*p*-value
			n (%)	n (%)	
		n (%)	3473 (66.1)	1784 (33.9)	
Area of Residence^^※^1^	Fukushima	734 (14.0)	521 (71.0)	213 (29.0)	0.002
	Hokkaido	523 (10.0)	337 (64.4)	186 (35.6)	0.407
	Tohoku	523 (10.0)	340 (65.0)	183 (35.0)	0.591
	Kanto	633 (12.0)	401 (63.3)	232 (36.7)	0.124
	Chubu	633 (12.0)	431 (68.1)	202 (31.9)	0.251
	Kinki	633 (12.0)	413 (65.2)	220 (34.8)	0.642
	Chugoku	525 (10.0)	326 (62.1)	199 (37.9)	0.043
	Shikoku	526 (10.0)	337 (64.1)	189 (35.9)	0.308
	Kyushu/Okinawa	527 (10.0)	367 (69.6)	160 (30.4)	0.068
Sex	Female	2512 (47.8)	1672 (48.1)	840 (47.1)	0.467
	Male	2745 (52.2)	1801 (51.9)	944 (52.9)	
Age	10–29	760 (14.5)	371 (10.7)	389 (21.8)	<0.001
	30–39	842 (16.0)	480 (13.8)	362 (20.3)	
	40–49	819 (15.6)	519 (14.9)	300 (16.8)	
	50–59	870 (16.5)	607 (17.5)	263 (14.7)	
	60–69	851(16.2)	622 (17.8)	229 (12.8)	
	70–79	736 (14.0)	583 (16.8)	153 (8.6)	
	80–99	379 (7.2)	291 (8.4)	88 (4.9)	
Received information on radiation and its health effects	Yes	1266 (24.1)	1074 (30.9)	192 (10.8)	<0.001
	No	3991 (75.9)	2399 (69.1)	1592 (89.2)	
Has attended a lecture on radiation health effects	Yes	705 (13.4)	590 (17.0)	115 (6.4)	<0.001
	No	4552 (86.6)	2883 (83.0)	1669 (93.6)	
Interested in topics related to the disaster	Yes	1076 (20.5)	984 (28.3)	92 (5.2)	<0.001
	Probably yes	2568 (48.8)	2031 (58.5)	537 (30.1)	
	Probably no	979 (18.6)	373 (10.7)	606 (34.0)	
	No	634 (12.1)	85 (2.4)	549 (30.8)	
Interested in topics related to the environment	Yes	990 (18.8)	924 (26.6)	66 (3.7)	<0.001
	Probably yes	2400 (45.7)	1957 (56.3)	443 (24.8)	
	Probably no	1146 (21.8)	474 (13.6)	672 (37.7)	
	No	721 (13.7)	118 (3.4)	603 (33.8)	
Considered that the FDNPP accident will result in intergenerational radiation health effects	Yes	1229 (23.4)	969 (27.9)	260 (14.6)	<0.001
	Probably yes	2124 (40.4)	1560 (44.9)	564 (31.6)	
	Probably no	1356 (25.8)	794 (22.9)	562 (31.5)	
	No	548 (10.4)	150 (4.3)	398 (22.3)	
Would like to visit the area around the FDNPP	Yes	446 (8.5)	396 (11.4)	50 (2.8)	<0.001
	Probably yes	1065 (20.3)	859 (24.7)	206 (11.5)	
	Probably no	2158 (41.1)	1447 (41.7)	711 (39.9)	
	No	1588 (30.2)	771 (22.2)	817 (45.8)	
Interested in the recovery of the area surrounding the FDNPP	Yes	1149 (21.9)	1056 (30.4)	93 (5.2)	<0.001
	Probably yes	2270 (43.2)	1826 (52.6)	444 (24.9)	
	Probably no	1224 (23.3)	482 (13.9)	742 (41.6)	
	No	614 (11.7)	109 (3.1)	505 (28.3)	
Accepting of the reuse of the soil in their current area or residence	Yes	490 (9.3)	406 (11.7)	84 (4.7)	<0.001
	Probably yes	1460 (27.8)	1133 (32.6)	327 (18.3)	
	Probably no	1654 (31.5)	1057 (30.4)	597 (33.5)	
	No	1653 (31.4)	877 (25.3)	776 (43.5)	

Yes/No on the table means whether they want more information about soil recycling resulting from the FDNPP accident in the Chi-squared test, FDNPP, Fukushima Daiichi Nuclear Power Plant; Chi-square test for each region^^※^1^.

Considering the number of respondents who wanted more information, their ages were distributed from lows of 8.4% (n = 291) in the 80s–90s and 10.7% (n = 371) in the teens–20s groups, to a high of 17.8% (n = 622) in the 60s cohort.

A majority of respondents (75.9%, n = 3991) reported that they had not received information on radiation and its health effects. Of the 24.1% (n = 1266) who had received such information, 30.9% (n = 1074) wanted more information on soil recycling, compared to just 10.8% (n = 192) who did not want additional information (*p* < 0.001). On the question of whether survey respondents had attended a lecture about radiation health effects, a clear majority, 86.6% (n = 4552), responded no. Of those who attended a lecture, 17.0% (n = 590) expressed a desire for more information about soil recycling, compared with those who did not (6.4%, n = 115).

Regarding an interest in disaster-related topics, 69.3% (n = 3644) of respondents answered “yes” or “probably yes.” Further, a majority (64.5%, n = 3390) answered “yes” or “probably yes” that they were interested in environment-related topics. An overwhelming majority in these two affirmative groups wanted more information on soil recycling: those interested in knowing more on the disaster answered yes at a rate of 86.8% (n = 3015), while those wanting more on the environment answered yes at a rate of 82.9% (n = 2881). Both groups, when compared with those who desired no more information, had a significant difference of <0.001 in the chi-square test.

Almost two-thirds of the respondents (63.8%, n = 3353) considered the Fukushima Daiichi nuclear accident would result in intergenerational radiation health effects. Of that group, 72.8% (n = 2529) wanted more information about soil recycling. A majority (71.3%, n = 3746) of respondents would not, or probably would not, want to visit the area around the FDNPP. Of those who did not want to visit, 63.9% (n = 2218) wanted more information about soil recycling. A majority (65.1%, n = 3419) of respondents answered “yes” or “probably yes” when asked if they were interested in the recovery of the area surrounding the FDNPP. Of that group, 83.0% (n = 2882) wanted more information about soil recycling. In the FDNPP and radiation risk questions, there were significant differences between those who wanted to receive more information and those who did not (*p* < 0.001).

When asked if they were accepting of the reuse of soil in their current residential area, 62.9% (n = 3307) responded “no” or “probably no.” Of this group, 55.7% (n = 1934) wanted more information on soil recycling, and 77.0% (n = 1373) did not want more information (*p* < 0.001).

### Information sources’ relationship to wanting more information on soil recycling

The relationship between the sources of information used by survey respondents on the topic of radiation and its health effects and wanting more information on soil recycling are shown in Table 2.

**Table 2:** Relationships between exposure to information sources on radiation and its health effects and wanting more information on soil recycling.

	Overall n = 1266	Yes	No	*p*-value
	n (%)	n (%)	n (%)	
		1074 (84.8)	192 (15.2)	
Internet search engines	674 (53.2)	598 (55.7)	76 (39.6)	<0.001
TV programs and newspapers	658 (52.0)	581 (54.1)	77 (40.1)	<0.001
News sites	491 (38.8)	446 (41.5)	45 (23.4)	<0.001
Books and magazines	393 (31.0)	347 (32.3)	46 (24.0)	0.012
Parents, friends, teachers & acquaintances	214 (16.9)	184 (17.1)	30 (15.6)	0.346
Social networking sites	205 (16.2)	189 (17.6)	16 (8.3)	<0.001
Video streaming services	158 (12.5)	143 (13.3)	15 (7.8)	0.019
Computer applications	100 (7.9)	89 (8.3)	11 (5.7)	0.142
Other	48 (3.8)	38 (3.5)	10 (5.2)	0.179

Yes/No on the table means whether they want more information about soil recycling resulting from the FDNPP accident in the Chi-squared test, Response = Yes.

The number of survey respondents who had received information on radiation and its health effects was 1266. The sources from which respondents obtained information, from most to least frequent, were: internet search engines 53.2% (n = 674); TV programs and newspapers 52.0% (n = 658); news sites 38.8% (n = 491); books and magazines 31.0% (n = 393); parents/friends, teachers, and acquaintances 16.9% (n = 214); social networking sites 16.2% (n = 205); video streaming services 12.5% (n = 158); computer applications 7.9% (n = 100); and other 3.8% (n = 48). Among these sources, internet search engines (*p* < 0.001), TV programs and newspapers (*p* < 0.001), news sites (*p* < 0.001), books and magazines (*p* = 0.012), social networking sites (*p* < 0.001), and video streaming services (*p* = 0.019) showed significant differences related to wanting more information on soil recycling.

### Types of desired information on the reuse of recycled soil in the area where respondents currently live and those who wanted this information

Survey respondents who wanted more information on soil recycling in the area where they currently live opted for the following types of information, from most to least frequent: health effects 55.1% (n = 2899), effects on food and water 48.6% (n = 2556), soil recycling methods 45.8% (n = 2406), environmental effects 45.2% (n = 2376), radioactivity levels and monitoring methods 40.0% (n = 2101), intergenerational health effects 35.8% (n = 1883), information disclosure methods 30.8% (n = 1617), risks during disasters 23.9% (n = 1255), and other information 2.5% (n = 132) (Table 3). In all of the desired information categories, there were significantly different responses between those who wanted more information on soil recycling and those who did not (all *p* < 0.001).

**Table 3:** Relationship between the type of desired information on the reuse of recycled soil in the area where respondents currently live and those who wanted the information.

	Overall n = 5257	Yes	No	*p*-value
	n (%)	3473 (66.1)	1784 (33.9)	
		n (%)	n (%)	
Health effects	2899 (55.1)	2243 (64.6)	656 (36.8)	<0.001
Effects on food and water	2556 (48.6)	2012 (57.9)	544 (30.5)	<0.001
Recycling methods	2406 (45.8)	1892 (54.5)	514 (28.8)	<0.001
Environmental effects	2376 (45.2)	1878 (54.1)	498 (27.9)	<0.001
Radioactivity concentration and monitoring methods	2101 (40.0)	1754 (50.5)	347 (19.5)	<0.001
Intergenerational health effects	1883 (35.8)	1528 (44.0)	355 (19.9)	<0.001
Information disclosure methods	1617 (30.8)	1158 (33.3)	459 (25.7)	<0.001
Risks during disasters	1255 (23.9)	990 (28.5)	265 (14.9)	<0.001
Other information	132 (2.5)	39 (1.1)	93 (5.2)	<0.001

Yes/No on the table means whether they want more information about soil recycling resulting from the FDNPP accident in the Chi-squared test, Response = Yes.

### Independent factors related to wanting more information about the recycling of removed soil

Binary logistic regression analyses made clear the independent factors related to wanting more information on the recycling of removed soil. Those who were interested in the recovery of the area surrounding the FDNPP (OR=2.199, 95% CI: 1.977–2.449, *p* < 0.001), interested in environmental topics (OR=2.017, 95% CI: 1.766–2.303, *p* < 0.001), interested in disaster-related topics (OR=1.789, 95% CI: 1.568–2.042, *p* < 0.001), who considered the FDNPP accident will result in intergenerational radiation health effects (OR=1.393, 95% CI: 1.266–1.533, *p* < 0.001), who accepted the reuse of the soil in the current living area (OR=1.358, 95% CI: 1.236–1.492, *p* < 0.001), and who would like to visit the area around the FDNPP (OR=1.136, 95% CI: 1.022–1.264, *p* = 0.019) were independently associated with wanting more information on the recycling of removed soil. In our model, demographic variables such as sex, age, living areas, and received information on radiation and its health effects were not significantly related to wanting more information on soil recycling (Table 4).

**Table 4:** Independent factors related to wanting more information about the recycling of removed soil.

Variable	Unit	Odds Ratio (95% CI)	*p*-value
Interested in the recovery of the area surrounding the FDNPP	Yes/No	2.199 (1.977-2.449)	<0.001
Interested in environmental topics	Yes/No	2.017 (1.766-2.303)	<0.001
Interested in disaster-related topics	Yes/No	1.789 (1.568-2.042)	<0.001
Considered the FDNPP accident will result in intergenerational radiation health effects	Yes/No	1.393 (1.266-1.533)	<0.001
Acceptance of the reuse of the soil in their current residential area	Yes/No	1.358 (1.236-1.492)	<0.001
Would you like to visit the area around the FDNPP	Yes/No	1.136 (1.022-1.264)	0.019
Sex	Female/Male	1.126 (0.970-1.308)	0.119
Age	10-80s	0.965 (0.924-1.008)	0.113
Riving areas	Fukushima/ others	1.065 (0.850-1.334)	0.585
Received information on radiation and its health effects	Yes/No	0.975 (0.757-1.257)	0.848

Binary logistic regression analysis, reference; wanting more information about recycling removed soil, CI; confidence interval.

## Discussion

### Concerns about the recycling of contaminated soil

The results of this nationwide study showed that approximately 60% of respondents did not want recycled contaminated soil to be relocated to the area where they currently live. This is in line with an earlier study conducted in 46 prefectures, excluding Fukushima, which identified important factors related to the final disposal of contaminated soil from the Fukushima nuclear accident [15]. That study found the public wanted final disposal sites for the removed soil to be far from residential areas. These key findings through both studies spotlight the fundamental problem with recycling soil for final disposal where people live: the public does not want it.

Our study shows that, if recycled soil is to be used where respondents live, the information they most desire concerns how their health might be affected. Almost two-thirds of the respondents wanted information on health effects; more than half wanted information on the effects on food and water, environmental effects, and radiation information; and almost half wanted information on intergenerational health effects. This was not unexpected because, in previous studies, nuclear waste hazards have ranked among the top perceived public health risks [8,16], and about half of all long-term evacuees in Fukushima remain anxious about possible health effects stemming from radiation exposure and intergenerational health effects on the next generation [17]. In our study, almost two-thirds considered the Fukushima Daiichi nuclear accident will result in intergenerational health effects. The public’s health concerns, including intergenerational health effects, are primary dialogue areas that may help to maximize the potential of achieving community understanding and nationwide soil recycling.

### The importance of having the right information when forming attitudes toward soil recycling

Having already received information on radiation and its health effects was not an independent factor for wanting more information about the recycling of removed soil. Thus, whether respondents were previously informed about radiation and its health effects, or lack thereof, was not a determinant of whether they desire more information on soil recycling. Almost two-thirds of respondents wanted more information about soil recycling, with 75% of them not having previously received radiation or health information and 86% not having attended a lecture on those topics, suggesting large information gaps that may be mitigated through information outreach. Prior research into public attitudes toward the permanent disposal of contaminated soil from the FDNPP highlighted the importance of procedural fairness that includes transparency and the disclosure of information [8,15], both of which have long been associated with public acceptance of “Not in My Backyard” (NIMBY) hazardous facilities [18]. This study also seeks to understand public attitudes toward the permanent disposal of contaminated soil from the FDNPP, and proactive information dissemination incorporating what was learned in this study and others may improve disclosure perception and prompt increased community interest in acquiring knowledge of recycling removed soil.

Interest in the recovery of the area surrounding the FDNPP was an independent factor closely related to wanting more information about the recycling of removed soil. Moreover, interest in environmental and disaster-related topics were independent factors related to wanting more information about the recycling of removed soil. Prior research into public attitudes of people in the Kanto and Kansai regions of Japan toward the national policy of disposing of contaminated soil in areas outside of the Fukushima Prefecture showed that approval was positively related with a strong interest in the nuclear accident and knowledge of decontamination and national policies [15]. The connection between wanting more information about soil recycling and having an interest in nuclear accident topics is an area to explore for more effective risk communication and proactive information dissemination in the future. The explanation of how soil recycling decisions are made and what discourse is needed to effectively communicate with local communities and stakeholders at different levels are also important areas to explore.

### Sources for receiving information and wanting more information

We examined the information sources reported by respondents who had received information on radiation and its health effects and their relationship to wanting more information on soil recycling. The top two information sources were internet search engines and TV programs and newspapers. More than half of the respondents who listed these sources wanted more information on soil recycling. News sites and books and magazines were information sources for approximately one third of the respondents, and about one third of that group wanted more information on soil recycling. Our study results show that internet search engines may be effective sources of soil recycling information for the group wanting more information. The priority should be in promoting interest in soil recycling, and these results suggest that an effective way of achieving greater interest could be through the development of search engine tools so people can easily gather the type(s) of information they want.

### Implications

The results of this study help us to understand the informational factors that influence the Japanese public’s acceptance of the disposal of contaminated soil from the Fukushima Daichi nuclear accident outside of the Fukushima Prefecture by 2045. Although permanent disposal of recycled soil is required by law, the areas of concern expressed by the public that are noted in this study and in prior research accentuates the importance of facilitating informed decision making and a participatory approach to arrive at an implementable, sustainable, strategy for contaminated soil recycling. A fundamental result of this study is understanding what the public wants to know when faced with soil recycling where they live. The use of this knowledge can help government and community stakeholders to have productive dialogues with the public about soil recycling. Seven other prefectures have similar final soil disposal problems; Miyagi, Iwate, Ibaraki, Tochigi, Gunma, Saitama, and Chiba have stored contaminated soil locally, but have not yet found final disposal sites [8], although each has stated it would dispose of the soil within their prefecture. The lessons learned from Fukushima may help the other prefectures. In this study we seek to understand key factors associated with the public’s desire for more information about soil recycling, and we approach it from a national perspective and did not single out Fukushima. Going deeper into Fukushima’s responses, and for any other specific prefecture such as where demonstration projects are targeted, can be the subject of future research.

The timely disposal of contaminated soil outside of Fukushima Prefecture is required by law, but government efforts to use recycled soil in several communities outside of Fukushima in pilot projects have not been successful [19,20]. Our study identified the type of information that elicits a statistically significant positive response to soil recycling, and there is also research available on the social science mechanisms of soil recycling [14,18,21] that, when examined together with our study results, help to create a better understanding of how to inform and set up a decision making process with the public on soil recycling. The national government wants to achieve a consensus on community soil recycling, as doing so is one of the steps for achieving final disposal outside of the prefecture by 2045 [3]. That objective has not yet been met and there are major challenges to overcome before there is the creation of a waste management strategy accepted by stakeholders, and the acceptance of soil recycling at the prefectural and local community levels. A survey completed in early 2021 of all prefectural governors in Japan, except for Fukushima, demonstrated a universal lack of support for soil recycling [22]. The government is taking steps to devise a strategy for the soil recycling issue. The IAEA has assisted the MOE through a series of three meetings and advised on technical soil volume reduction and recycling topics, as well as on public communication and stakeholder engagement [5]. In December 2024, the government announced a planned cabinet meeting to expedite Fukushima soil disposal by clearly explaining its position on resolving the problem and expanding the number of ministries involved, such as the ministries of reconstruction, land, infrastructure, transport and tourism, agriculture, forestry and fisheries, and internal affairs and communication [23]. These efforts underscore the government’s position that Fukushima’s recovery and revitalization is a national priority [5,24]. However, despite the government’s efforts so far, this research and other recent studies, and the public’s opposition to demonstration projects point out the challenges that remain in meeting the legal requirement to move the recycled soil resulting from the FDNPP accident out of Fukushima Prefecture by 2045.

The unprecedented need to dispose of large amounts of contaminated soil in areas of Japan after a nuclear accident’s decontamination effort has, not unexpectedly, run up against an unwilling public that does not want the soil where they live and will not agree to demonstration projects. However, what we have learned from research in the past few years, and in the present study, gives some hope that, by implementing appropriate public engagement, effective risk communication, and information outreach, members of the public can achieve greater understanding of the soil recycling process and how they may or may not be personally impacted. Achieving this understanding is an important milestone. Community leaders and residents would very reasonably want to understand the costs and benefits, and why and in which conditions they should accept a waste management strategy including part of the recycled soil.

### Limitations of the study

This is a retrospective study that can only establish associations between factors, not causation. A web-based survey was conducted, and survey responses were collected by gender and age group based upon the population composition ratio of the Fukushima Prefecture, Tohoku, Kanto, and Kyushu. As such, the survey respondents may differ from regional populations in education levels, occupation, family demographics, and other factors.

## Conclusions

This study’s results explain what the public wants to know to better understand soil recycling and identify the sources and types of information that matter the most to them. Specific information is identified that can foster a greater understanding of soil recycling. Although a national policy to use Fukushima’s recycled soil throughout Japan has been announced, 75.9% of respondents nationwide have not received information on radiation or its health effects, and 86% of respondents have never attended a lecture on the topic. These large knowledge gaps must be addressed.

We learned that, when confronted with soil recycling in the areas where they live, respondents’ top priorities included obtaining more information on health effects, which was closely followed by effects on food and water, and then by environmental effects, levels of radiation and how radiation is monitored, and intergenerational health effects. Key independent factors related to wanting more information on soil recycling included interest in the disaster and surrounding area, interest in topics related to the environment and the disaster, a belief that the nuclear accident would result in intergenerational health effects, and the acceptance of the reuse of soil in one’s current residential area.

## Supporting information

S1 Appendix The questionnaire of the survey. (DOCX)

S1 Checklist PLOSOne Human Subjects Research Checklist. (DOCX)

## References

[1] Ministry of the Environment. Decontamination | Environmental remediation [Internet]. Government of Japan [cited 2024 Dec 11]. Available from: https://josen.env.go.jp/en/decontamination/

[2] Ministry of the Environment. Interim Storage Facility | Environmental Remediation [Internet]. Government of Japan [cited 2024 Dec 11]. Available from: https://josen.env.go.jp/en/storage/

[3] International Atomic Energy Agency. Japan’s Fukushima soil recycling and disposal plan meets safety standards, IAEA says. IAEA. 2024 Sep 10 [cited 2024 Dec 16]. Available from: https://www.iaea.org/newscenter/pressreleases/japans-fukushima-soil-recycling-and-disposal-plan-meets-safety-standards-iaea-says

[4] Mori Y. Progress in Off-site Environmental Remediation in Japan. Ministry of the Environment, Japan. 2024. Available from: https://www.meti.go.jp/english/earthquake/nuclear/decommissioning/pdf/241015e.pdf

[5] Ministry of the Environment. Publication of the final report of the IAEA experts meeting on volume reduction and recycling of removed soil arising from decontamination activities [Internet]. Government of Japan [cited 2024 Dec 12]. Available from: https://www.env.go.jp/en/press/press_03156.html

[6] Ministry of the Environment. What Does Recycling of Removed Soil Mean? [Internet]. Government of Japan [cited 2024 Dec 12]. Available from: https://josen.env.go.jp/chukanchozou/material/pdf/removed-soil_recycling-en_2205.pdf

[7] Watanabe S. Disposing of Fukushima’s Contaminated Soil. NHK World-Japan News. 2019 Mar 10 [cited 2024 Dec 16]. Available from: https://www3.nhk.or.jp/nhkworld/en/news/backstories/399/

[8] Slovic P. The perception gap: Radiation and risk. Bull At Sci. 2012;68(3):67–75. doi: 10.1177/0096340212444870

[9] Gupta K, Ripberger J, Fox A, Maiello M, Peach K, Jenkins-Smith H. Risk communication and public response to potential radiation emergencies in New York City. Risk Analysis. doi: 10.1111/risa.17657 39380450

[10] Takada M, Murakami M, Ohnuma S, Shibata Y, Yasutaka T. Public Attitudes toward the Final Disposal of Radioactively Contaminated Soil Resulting from the Fukushima Daiichi Nuclear Power Station Accident. Environ Manage. 2024;73(5):962–72. doi: 10.1007/s00267-024-01938-w 38305854 PMC11023960

[11] Shirai K, Takada M, Murakami M, Ohnuma S, Yamada K, Osako M, et al. Factors influencing acceptability of final disposal of incinerated ash and decontaminated soil from TEPCO’s Fukushima Daiichi nuclear power plant accident. J Environ Manage. 2023;345:118610. doi: 10.1016/j.jenvman.2023.118610 37536131

[12] Yokoyama M, Ohnuma S, Osawa H, Ohtomo S, Hirose Y. Public acceptance of nuclear waste disposal sites: A decision-making process utilising the veil of ignorance concept. Humanit Soc Sci Commun. 2023;10(1):1–10. doi: 10.1057/s41599-023-02139

[13] Yasumura S, Ohira T, Ishikawa T, Shimura H, Sakai A, Maeda M, et al. Achievements and Current Status of the Fukushima Health Management Survey. J Epidemiol. 2022;32(Suppl_XII):S3–10. doi: 10.2188/jea.JE20210390 36464298 PMC9703928

[14] Orui M, Nakayama C, Kuroda Y, Moriyama N, Iwasa H, Horiuchi T, et al. The Association between Utilization of Media Information and Current Health Anxiety Among the Fukushima Daiichi Nuclear Disaster Evacuees. Int J Environ Res Public Health. 2020;17(11):3921. doi: 10.3390/ijerph17113921 32492886 PMC7312024

[15] Takada M, Shirai K, Murakami M, Ohnuma S, Nakatani J, Yamada K, et al. Important factors for public acceptance of the final disposal of contaminated soil and wastes resulting from the Fukushima Daiichi nuclear power station accident. PLoS One. 2022;17(6):e0269702. doi: 10.1371/journal.pone.0269702 35731732 PMC9216558

[16] Flynn J, Slovic P, Mertz CK. Gender, race, and perception of environmental health risks. Risk Anal. 1994;14(6):1101–8. doi: 10.1111/j.1539-6924.1994.tb00082.x 7846319

[17] Hande V, Orita M, Matsunaga H, Kashiwazaki Y, Taira Y, Takamura N. Changes in the Intention to Return and the Related Risk Perception Among Residents and Evacuees of Tomioka Town 11 Years After the Fukushima Nuclear Accident. Disaster Med Public Health Prep. 2023;17:e386. doi: 10.1017/dmp.2023.58 37165606

[18] Cotton M. Environmental Justice as Scalar Parity: Lessons From Nuclear Waste Management. Soc Just Res. 2018;31(3):238–59. doi: 10.1007/s11211-018-0311-z

[19] Takeda R. Tokyo, Saitama Residents Say no to Living Near Fukushima Soil. Asahi Shimbun. 2023 Feb 25 [cited 2024 Dec 8]. Available from: https://www.asahi.com/ajw/articles/14848079

[20] Sekine S. Ministry to Delay Trial on Reusing Fukushima Soil in Kanto Areas. Asahi Shimbun. 2023 Mar 2 [cited 2024 Dec 8]. Available from: https://www.asahi.com/ajw/articles/14851976

[21] Maxwell K, Kiessling B, Buckley J. How clean is clean: a review of the social science of environmental cleanups. Environ Res Lett. 2018;13(8):10.1088/1748-9326/aad74b. doi: 10.1088/1748-9326/aad74b 32076455 PMC7029711

[22] Fukuchi K, Toda M. Survey: Not a single prefecture backs reuse of radioactive soil. Asahi Shimbun. 2021 Mar 28 [cited 2024 Dec 14]. Available from: https://www.asahi.com/ajw/articles/14311546

[23] Yomiuri Shimbun. Japan Cabinet to discuss disposal of Fukushima soil; government plans to expand scope of ministries involved. Japan News. 2024 Dec 5 [cited 2024 Dec 12]. Available from: https://japannews.yomiuri.co.jp/society/general-news/20241205-226032/

[24] Voice of America. New Japan Cabinet convenes; serious challenges ahead [Internet]. 2011 Dec 1 [cited 2024 Dec 13]. Available from: https://www.voanews.com/a/new-japan-cabinet-convenes-to-confront-serious-challenges-129043993/144686.html

