# Correction: What the public wants to know about the recycling of contaminated soil

**DOI:** 10.1371/journal.pone.0347369

**Published:** 2026-04-14

**Authors:** Stephen Takeshi Terada, Hitomi Matsunaga, Aizhan Zabirova, Tomoko Watanabe, Yuya Kashiwazaki, Makiko Orita, Thierry Schneider, Noboru Takamura

The third author’s name is spelled incorrectly. The correct name is: Aizhan Zabirova. The correct citation is: Terada ST, Matsunaga H, Zabirova A, Watanabe T, Kashiwazaki Y, Orita M, et al. (2025) What the public wants to know about the recycling of contaminated soil. PLoS One 20(9): e0331478. https://doi.org/10.1371/journal.pone.0331478.
