# Supplementary material for: What the public wants to know about the recycling of contaminated soil
Source: PLoS One. 2025 Sep 4;20(9):e0331478. doi: 10.1371/journal.pone.0331478 (PMC12410821; doi:10.1371/journal.pone.0331478)
Supplement: S1 Appendix — (DOCX) [file pone.0331478.s001.docx]

"Survey on Awareness of Recycling of Contaminated Soil from Decontamination

after the Fukushima Daiichi Nuclear Power Plant Accident"

Please check the appropriate box and complete the applicable items in ( ).

1. Area of residence

□Hokkaido, □Fukushima, □Tohoku, □Kanto, □Chubu, □Kinki, □Chugoku, □Shikoku, □Kyushu/Okinawa.

1. Gender

□Female □Male

1. Age

( ) years old

1. Have you ever collected information on radiation and its health effects?

□Yes □No

　 　↓

1. For those who answered "yes." What method did you use to collect information?

(Multiple choice)

□Books/magazines □Asked by such as Parents, friends, teachers & acquaintances □TV programs/newspapers □Internet search engines □Social networking sites □Video streaming services □News site □Computer applications □Other ( )

1. Have you ever attended radiation and its health effects during lectures or talk?

□Yes □No

1. Are you interested in disaster-related topics?

□Yes □Probably yes □Probably no □No

1. Are you interested in topics related to environmental issues?

□Yes □Probably yes □Probably no □No

1. Do you think that the Fukushima Daiichi Nuclear Power Plant accident will cause the health effects of radiation to the next generation?

□Yes □Probably yes □Probably no □No

1. Would you like to visit the area around the Fukushima Daiichi Nuclear Power Plant?

□Yes □Probably yes □Probably no □No

1. Are you interested in the recovery of surrounding areas after the Fukushima Daiichi Nuclear Power Plant accident?

□Yes □Probably yes □Probably no □No

1. Do you think it is acceptable to reuse the soil in your current area or residence?

□Yes □Probably yes □Probably no □No

1. What type of desired information is available regarding the reuse of recycled soil in your area? (Multiple choice)

□Information disclosure methods □Risks during disasters □Health effects

□Radioactivity concentration and monitoring methods □Environmental effects □Effects on food and water □Intergenerational health effects

That is, all the questions I have. Thank you for your comment.

＊＊＊＊＊＊＊＊＊＊＊＊＊＊＊＊＊＊＊＊＊＊＊＊＊＊＊＊＊＊＊＊＊＊＊＊＊＊＊＊＊＊＊＊
